# Assessment of the role of *Trichomonas tenax* in the etiopathogenesis of human periodontitis: A systematic review

**DOI:** 10.1371/journal.pone.0226266

**Published:** 2019-12-17

**Authors:** C. Bisson, S. M. Dridi, M. Machouart

**Affiliations:** 1 Stress, Immunity, Pathogens Laboratory, EA7300 Lorraine University, Faculty of Medicine, Vandoeuvre- lès- Nancy, France; 2 CHU Odontologie de Nancy - Department of Periodontology– Rue du Docteur Heydenreich, Nancy, University Lorraine, France; 3 CHU Odontologie de Nice -Department of periodontology- CHU Saint Roch – Department of Periodontology, Nice, France; 4 CHU Brabois, Parasitology - Mycology Laboratory, CHU Nancy-Brabois, Vandoeuvre-les -Nancy, France; New York Medical College, UNITED STATES

## Abstract

**Objective:**

This systematic review was to assess the presence of *Trichomonas tenax* in patients with periodontitis and to elucidate its potential role in the onset and development of this disease.

**Method:**

Systematic review was conducted according to the Preferred Reporting Items for Systematic Reviews and Meta-Analyses (PRISMA) guidelines and by consulting the five databases: Medline, Science Direct, Web of Science, Dentistry and Oral Science Sources and Cochrane Central Register of Controlled Trials. Following Koch’s postulates revisited by Socransky as PICO framework, this collection data was only including full text of clinical trials concerning patients with periodontitis, case-reports and in vitro research published between 1960 and March 2019.

**Results:**

On the 376 studies identified, only 25 fulfilled our eligible criteria. Most of these studies were *in vitro* research articles designed to evaluate potential virulence factors, and others were clinical trials (case-control studies, randomized controlled trial) and case-reports. The analysis of these papers has shown that i) *Trichomonas tenax* is more frequently detected in dental biofilm from sites with periodontitis than in healthy sites; ii) this live flagellate seems capable of producing diverse enzymes that could participate in periodontal breakdown and has the capacity to adhere to epithelial cells, its lysed form could induce the synthesis of IL-8 from macrophage cell lines; iii) the impact of non-surgical treatment of periodontitis have not been thoroughly evaluated on the presence of *T*. *tenax*

**Conclusions:**

This systematic review has reported the presence of *T*. *tenax* more frequently in diseased than healthy sites and the capacity of this flagellate to synthesis enzymes which could participate to the degradation of periodontal tissues. Nevertheless, these data do not meet all the postulates and are not enough to provide firm conclusions about the role of *T*. *tenax* in the etiopathogenesis of periodontitis.

## Introduction

The global burden of oral disorders and their associated sequelae have been assessed at various dates (1990, 2005 and 2010) by Marcenes et al. [1]. These authors concluded that DALYS (Disability-Adjusted Life Years) induced by oral conditions had increased by 20.8% between 1990 and 2010 due to population growth, the ageing process and severe periodontitis. Severe periodontitis is the sixth most prevalent disease affecting millions of people worldwide and because of its dental and systemic impacts should therefore be a public health priority [2].

Periodontitis is recognized as an inflammatory disease, mainly induced by pathobionts such as *Porphyromonas gingivalis* in synergy with other putative pathogens present in a complex and diversified biofilm. These periodontopathogens cause an impaired immune response leading to the loss of periodontal tissue [3]. But neither immune deficiencies nor the virulence of periodontal pathogens can explain why, despite the identification of periodontal pathogenic bacteria in the saliva, only a few teeth are severely affected by periodontitis in patients, or why one tooth presented alveolar bone and soft tissue destruction while the periodontium of its neighbour was barely affected.

In order to explain these clinical observations, but also the failure in stabilizing periodontitis in certain patients, the authors attempted to explore other potential etiologies of periodontal diseases. The oral cavity harbours a rich and complex biofilm that includes bacteria, but also viruses, fungi and protozoans [4]. All these microorganisms could be implicated directly or indirectly in the development of periodontitis. A systematic review with meta-analysis evaluating the microbiota from patients with periodontitis has shown a significant presence of some herpesviruses and other authors have identified fungi (*Candida* genus), both of which could have a potential pathological role in this disease [5,6]. Moreover, two main protozoans have been identified in the oral cavity: *Entamoeba gingivalis* belonging to the rhizopod class and *Trichomonas tenax* to the zoomastigophora one. As regards *Trichomonas tenax*, some authors suggested its implication in periodontal diseases [7, 8]. A recent review concerning *T*. *tenax* and periodontal diseases has been published in which the authors concluded that there was a correlation between the presence of the flagellate and the periodontal diseases and that this protozoan was involved in inflammatory process of gingivitis [9]. Nevertheless, this review did not follow the PRISMA (Preferred Reporting Items for Systematic Reviews and Meta-Analyses) guidelines and the PICO (Patient Intervention Comparison Outcome) framework as recommended by the Centre for Evidence-Based Medicine [10, 11]. In this review, 47 clinical studies about prevalence of the protozoan in periodontal diseases were not classified according to population (adults, children), presence of systemic disease, previous antibiotic consumption, and the keywords used for the literature research on periodontal diseases assessment were not detailed.

Therefore, we propose here the first systematic review of literature to investigate the presence of *T*. *tenax* in periodontitis in adult patients and to assess its potential role in the etiopathogenesis of periodontitis, according to Koch’s postulates revisited by Socransky [12]: (i) postulate “Association” (elevated level of microorganism in lesions of periodontitis), (ii) postulate “Elimination” (microorganism elimination resulted in successful therapy), (iii) postulate “Host response” (microorganism induces immune response: elevated serum and local antibodies, cytokines production), (iv) postulate “Virulence factors” (microorganism produces enzymes inducing a periodontal degradation) and (v) postulate “Animal studies” (microorganism induces disease in gnotobiotic animal).

Firstly, we investigate through literature data the presence of *T*. *tenax* in diseased sites and its absence in healthy sites in adult patients with periodontitis, secondly the impact of non-surgical periodontal treatment on the decrease or elimination of this flagellate in healed sites and thirdly the potential virulence factors of this protozoan which could cause the destruction of periodontal tissue.

## Methods

### Protocol and study design

This systematic review was elaborated by following the PRISMA guidelines [10] (S1 Table).

### Eligibility criteria

In this review, the focused questions addressed using the PICO framework, aimed to evaluate the potential pathogenicity of *Trichomonas tenax* in the development of periodontitis according to Koch’s postulates revisited by Socransky [12]:

1. Is *T*. *tenax* (I) identified in high numbers in sites with periodontitis (P) and in lesser numbers or not at all in healthy sites? (postulate “Association”)
2. Does the non-surgical treatment of periodontitis induce the decrease or the absence of *T*. *tenax* in the healed periodontal pocket and/or periodontal pocket with depth reduction? (C) (postulate “Elimination”)
3. Does *T*. *tenax* produce virulence factors (adhesion capacity and tissue invasion, immunomodulating molecules, toxicity) and can it induce similar periodontal diseases in an appropriate animal model? (O) (postulates “Virulence factors”, “Host response” and “Animal studies”).

### Information sources and research strategy for relevant studies

The five databases MEDLINE, Science Direct, Web of Science, Dentistry and Oral Sciences Source and Cochrane Central Register of Controlled Trials were consulted and our research was conducted on all relevant literature up to March 2019 using MeSH, and the following keywords: *Trichomonas tenax* AND (periodontal diseases), Trichomonas tenax AND (periodontitis), *Trichomonas tenax* AND (antibody) OR (pathogenicity) OR (virulence) OR (enzyme) OR (cytokines) OR (toxicity) OR (adhesion) OR (adherence) OR (invasion) OR (invasive).

No publication was selected prior to the 1960s, because terms used to evaluate periodontal status were confusing (pyorrhoea, dying periosteum…). The electronic research was conducted independently by two authors (CB, SMD).

### Study selection

Only full text articles published in the English, French, Italian and Spanish/Portuguese languages were considered.

All studies relating to *Trichomonas tenax* were considered eligible for inclusion if they were original articles in peer-reviewed journals conducted in humans and were:

- Cross sectional, case-control studies, controlled trials
- Case reports or case series (accepted for research pathogenicity factors).
- *In vitro* studies with evaluation of potential virulence factors and animal models of periodontitis.

All reviews of literature were excluded from the selected articles because they give redundant informations and are not detailed enough.

#### Inclusion criteria

Studies that were selected for this analysis include the following criteria:

- Adult population (≥ 18 years)
- Patients diagnosed with periodontitis using one or more of the following terms: pocket > 3 mm and/or CAL > 2 mm (Clinical Attachment Level), bone loss, grade of the periodontal tissue degradation (moderate or severe periodontitis, Armitage classification, 1999) [13].
- Patients diagnosed with periodontitis with no surgical treatment in the last 3 months (to evaluate the prevalence of *T*. *tenax* in periodontal pockets)
- Patients diagnosed with periodontitis treated by non-surgical treatment (to evaluate the impact of the therapy on protozoan in the healed sites).
- Studies with collection of dental biofilm (supra and/or subgingival)
- *In vitro* studies with detailed protocol

#### Exclusion criteria

Articles were excluded from the selection because of the presence of:

- Adolescent and children population (< 18 years)
- Unclear periodontal diagnosis of patients included, no precise description of periodontal parameters
- Periodontitis as manifestations of systemic disease
- Patients who received surgical periodontal therapy
- Patients taking medication that could modify the oral microbiota
- Studies relating only to the collection of saliva, tooth decay or oral mucosa.
- *In vitro* studies with other trichomonads species than *T*. *tenax*.

### Data selection process and elements

Data were collected independently by two authors (CB, SMD) and definitively included by these two authors, and all disagreements were resolved after discussion and/or with the intervention of a third author (MM). Reasons of exclusion of the articles were fully detailed in S1 Table, and quality appraisal of included clinical studies was described in S2 Table.

If the study was published twice, the article presenting the most complete data set was selected.

All publications meeting the eligibility criteria were included, processed for data extraction and categorized according to the PICO framework:

**Population**: adult patients with periodontitis

**Intervention**: presence of trichomonads in sites with periodontitis

**Comparison**: absence or reduction of the flagellates in diseased sites after non-surgical treatment

**Outcome**: potential virulence factors of the protozoan (adhesion, tissue invasion, cytokines, antibody, toxicity).

### Risk of bias in individual studies and across studies

Risk of bias was assessed through the Newcastle-Ottawa Scale (NOS) for case-control studies and modified NOS for cross-sectional studies [14]. The following criteria were assessed:

- Representativeness of the exposed cohort
- Ascertainment of exposure
- Quality of measurements/protocol and outcomes: periodontal parameters and precise protocol of collection of biofilm are evaluated.

For *in vitro* research articles, protocols [time of incubation, strain of *T*. *tenax*, different MOI (Multiplication of Infection), different cells line] used by each author are detailed.

## Results

### Study selection

**Fig 1:**
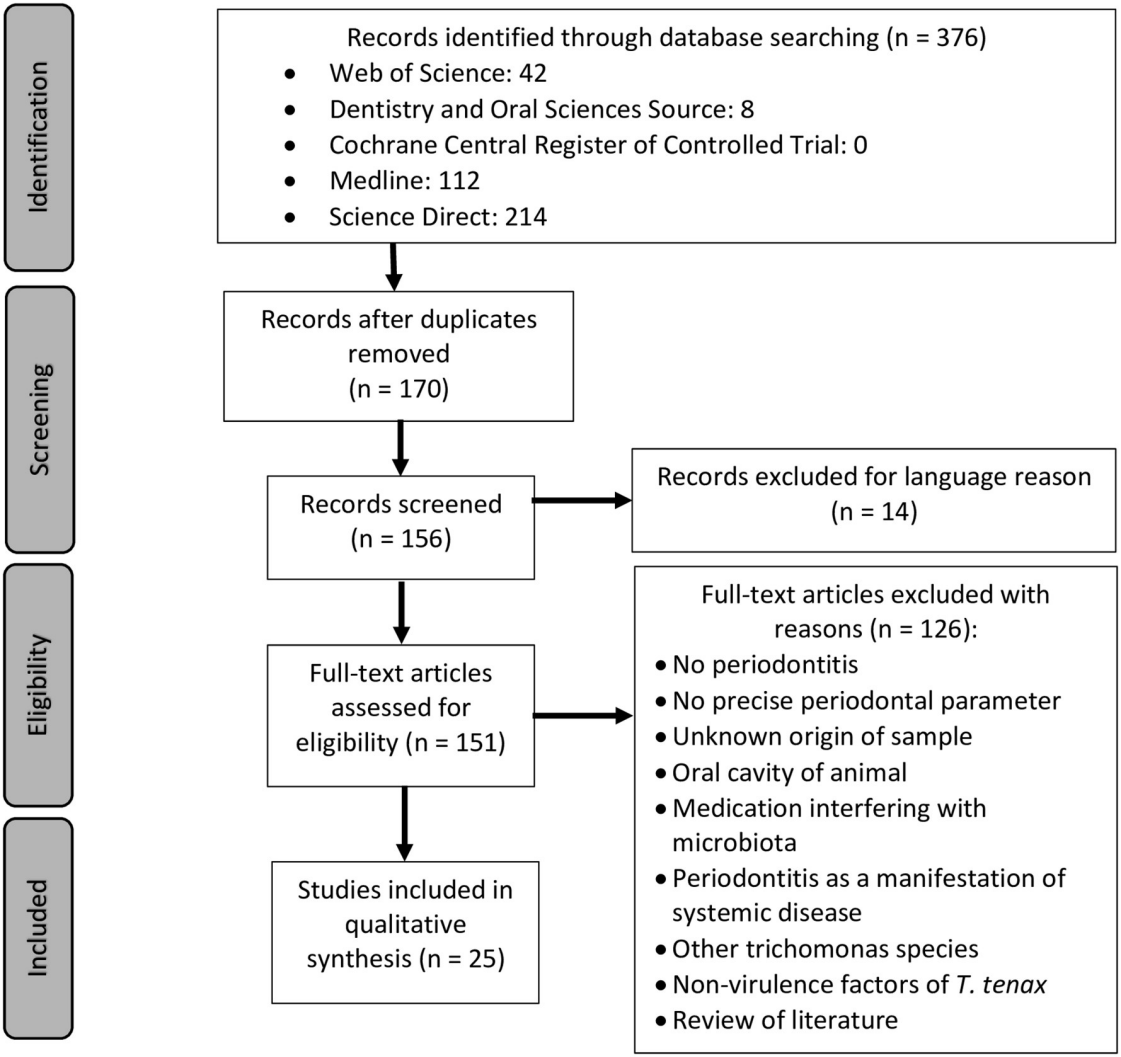
Study flow diagram.

As shown in Fig 1, a total of 376 articles were identified from the previously described electronic database search. After duplicate removal and language exclusion, a total of 156 articles were obtained. Finally, after the removal of non-full text and non-relevant papers, 25 articles were deemed appropriate to be included. The main reasons for exclusion were: the absence of periodontal status, lack of precise periodontal parameters, the identification of trichomonads in animal studies but not in human studies, the studies included patients with medication modifying microbiota, the diagnosis of periodontitis as a manifestation of systemic disease, other trichomonads species than *T*. *tenax*, the absence of a *T*. *tenax* factor of virulence. Some clinical studies were discarded because essential details were missing:

1. One study included patients with periodontitis as well as patients with periodontitis and diabetes and did not specify the proportion of patients *T*. *tenax*-positive among those with diabetes.
2. one study includes both children and adults but did not specify the *T*. *tenax* positivity according to the population age.
3. In other studies, some included patients had already been treated with antibiotics, without anyone knowing the proportion of patients *T*. *tenax* positive among those receiving or not antibiotics.
4. Finally, in some papers the origin of the sample was unknown such as “rinsing sample” and/or samples from saliva, mucosa, decay.

### Characteristics of included studies

- **Presence of *T*. *tenax* in periodontitis or** gingivitis sites and in healthy sites from controls (Table 1). Three case-control studies were selected to evaluate the presence of *T*. *tenax* in periodontitis (Table 1). Two of them have used the microscope to detect the flagellates, and only one the molecular biology tools. Whatever the technique used, the authors showed a higher frequency of this flagellate in patients with periodontitis (from 42.5 to 32.3%) than those with gingivitis (from 32.3% to 0%). The controls with healthy periodontium had positivity to *T*. *tenax* from 28.2% to 7.1%. The frequency of the flagellate in the control group doubled depending on authors, but its frequency in periodontitis sites was similar [7,8,15].
- **Impact of non-surgical treatment of flagellate** in healed sites (Table 1). Only one randomized controlled trial evaluated the impact of scaling and root planing (SRP) on the frequency of *T*. *tenax* in periodontitis sites [16]. The authors selected patients with moderate to severe periodontitis (CAL > 5 mm) and randomly assigned them to the experimental and control group. Before receiving an oral hygiene instruction, a sample of dental biofilm was collected from the patients in each group. Three weeks after SRP, a second biofilm sample was collected from patients in the experimental group. The authors concluded that the SRP could reduce the number of *T*. *tenax* in saliva but not in the dental plaque of patients with moderate to severe periodontitis. No detailed description of PPD (Periodontal Pocket Depth) and CAL before and after SRP was reported.
- **Potential virulence factors from protozoans** (Table 2). Authors have detected the capacity of *T*. *tenax* ATCC 30207 to adhere to diverse cells and induce cytotoxicity [17], while others studies showed no adherence or cytotoxic property [18–19]. Moreover, flagellates produce numerous enzymes, such as cysteine protease, cathepsin B like protein, haemolysins, metalloproteinase and other proteinases that could participate in the breakdown of collagen, gelatin observed in periodontal diseases [20–25]. Live trophozoites of the Hs-4 strain of *T*. *tenax* failed to induce secretion of IL-1β, IL-8, IL-10 or TNFα from macrophages. Lysed trophozoites stimulated synthesis of IL-8 from phagocytic cells [26]. The intravenous injection of *T*. *tenax* antigen and/or live flagellates in the rabbit elicits the production of antibodies [27,28]. The observation by transmission electron microscopy revealed the presence of bacteria inside the *T*. *tenax* cytoplasm: some were dividing while others were dead.
- **Research on the potential pathogenicity and invasive power of *T*. *tenax* in extra-oral location** (Table 3). Flagellates have been isolated from sputum, pleural sample, bronchoalveolar lavage, cervical lymph nodes, vaginal swabs, pharyngeal samples, urines and subhepatic abscess pus [29–35]. Generally, the identification was a microscope observation with staining or PCR. The authors concluded an aspiration of the parasite from the oral cavity to the lung location, and autoinoculation via hands or through oral sex for vaginal and urine identification. However, in two case reports, *T*. *tenax* could have migrated via the lymphatic vessels to the cervical lymph node and via bloodstream to subhepatic abscess. Some authors suggested a potential pathogenic role and/or flagellate co-infection agent in various infections. But most of these publications are case reports presenting infectious disease in immunocompromised patients.

**Table 1:** Identification of *T*. *tenax* in patient with or without periodontal disease.

Postulates	Authors Country Study design	Population description Sample size (N) Diagnosis periodontal disease	Criteria to define periodontal status	Selection and identification methods Samples	Results: positivity to T. tenax	Outcomes—Limitations
**Association**	Benabdelkader et al. 2019 [8] France Case-control study	*191 patients (19 ≤ years≤87, 95 males and 96 females): -control (Heathy gum, N = 85) -Periodontitis (N = 106)	*No information on periodontal indexes. * Diagnosis of periodontitis according to probing depth and attachment loss (Mild, N = 19; Moderate, N = 27, severe, N = 60)	* Pool of multiple sub gingival plaques collection per patient * qPCR^c^ from direct detection of collected sample or after culture of the collected sample.	* Culture qPCR result: -34.9% periodontitis patients -18.8%% control patients *Direct qPCR result -34% periodontitis patients -28% control patients *Combination of both results -42.5% periodontitis patients -28.2% control patients	Outcomes: more frequent detection of *T*. *tenax* in patients with periodontitis than healthy patients. Limitations: * Periodontitis patients: no trichomonads positivity according to PPD^a^ and CAL^b^ parameters
	Ferrara et al. 1986 [15] Italy Case-control study	*59 patients (25 ≤ years≤60, 29 males and 30 females): -Heathy gum (N = 14) -Gingivitis (N = 14) -Periodontitis (N = 31)	*No information on periodontal indexes. * Diagnosis of periodontal disease according to the tooth supporting tissue: Health, superficial (gingivitis) or profound inflammation (periodontitis)	*1 Supra and 1 sub gingival plaque per patient * Microscope observation of culture after incubation of 6 days.	* 18,6% all patients * 7,1% healthy patients *0% gingivitis patients *32,3% periodontitis patients	Outcomes: patients T. tenax positive: only periodontitis and healthy patients. Limitations: * Periodontitis patients: no description of PPD^a^ and CAL^b^ parameters * No control of flagellate species by PCR^c^
	Feki et al. 1981 [7] France Case-control study	* 300 patients (15 ≤ years ≥65) *Periodontal status: -healthy periodontium (N = 100) -gingivitis (N = 96) -periodontitis (N = 104)	*No information on periodontal indexes *Diagnosis of: -Periodontitis = bone resorption, pocket -Gingivitis = bleeding, modifications of colour and aspect of gingiva. -Health = no modification	* One sample of dental plaque, tartric sample per patient *Microscope observation of culture after incubation of 48 hours.	*28,7% all patients (19 < years≤65) *13% patients with healthy gum *32,3% patients with gingivitis *38,5% patients with periodontitis	Outcomes: highest percentage of T. tenax for periodontitis patients, followed by gingivitis and healthy patients. Limitations: *Periodontitis patients: no description of PPD^a^ and CAL^b^ parameters *Only inclusion of patients consulting Hospital Dental Department *Matching of sample size of the periodontitis, gingivitis and healthy groups, but no information for age and sex group. * No control of flagellate species by PCR^c^
**Elimination**	Rashidi Maybodi et al.2016 [16] Iran Randomized Clinical trial	46 patients (30<years<50) * case group (15 with severe and 8 with moderate periodontitis) *control group (14 with severe and 9 with moderate periodontitis) *exclusion: previous consumption of antibiotics, immunomodulators	*Moderate (CAL = 3–4 mm) to severe (CAL≥ 5 mm) periodontitis (classification of Armitage, 1998). *CAL of case group: 5.74 ± 1.7mm *CAL of control group: 5.64 ± 1.7mm	For each patient, collection of: 1 dental plaque and 1 saliva samples *For control group, samples collected before OHI *For case group, samples collected before OHI and 3 weeks after SRP. * Giemsa staining and microscope observation of sample	* Baseline frequency (%) of *T*.*tenax* for both group: not reported *Correlation between severity of periodontitis and frequency of *T*.*tenax* in dental plaque: r = 0.565, p≤0.005) *Identification of *T*.*tenax* only in dental plaque of female *3 weeks after SRP, significant reduction of *T*.*tenax* frequency in saliva but not in dental plaque.	Outcomes: CAL^b^ is representative of the degree of degradation of periodontal tissues Limitations: *No assessment of outcome, no description of periodontal indexes (except for CAL^b^), no report of correlation between periodontal parameters and T. tenax presence. *No report of blindness of data collection *Only inclusion of patients consulting School of Dentistry. * No information on level of infection (PPD^a^ not reported). *No control of flagellate species by PCR^c^

PPD^a^: Periodontal Pockets Depth,

CAL^b^: Clinical Attachment Level,

PCR^c^: Polymerase Chain Reaction/

qPCR^c^: quantitative PCR.

**Table 2:** Potential virulence factors of *T*. *tenax*.

Postulates	Authors	Experiment	Results	Outcomes—Limitations
**Virulence factors** Adhesion, Tissue invasion, Cytotoxicity	Ribeiro *et al*. 2015 [17]	*Adhesion *T*. *tenax* ATCC^a^ 30207 (axenic medium)—mammalian cell monolayer (epithelial cells, HeLa^e^, MDCK^c^) (SEM study). *Cytotoxicity assays (MOI 5:1, flagellates: cells HeLa, MDCK) MTT^b^ evaluation. *Interactions (MOI^h^ 1:1) *T*. *tenax*- 3D spheroid (epithelial cells and fibroblasts) SEM^f^ study, during 2,6,12 & 24h.	*Significant time dependent *T*. *tenax* cytotoxicity on MDCK^c^ and HeLa^e^ viability. * Significant damage of 3D spheroid by *T*. tenax * 3D spheroid penetration of *T*. tenax *Damage and disrupting of MDCK^c^ and HeLa^e^ monolayers by *T*. *tenax*. * Adhesion of *T*. *tenax* on different cells (MDCK^c^, HeLa, gum cells, 3D spheroid)	**Outcomes**: capacity to adhere on different cells, to penetrate inside 3D spheroids and to induce cytotoxic effects and damages of cells monolayer (disrupting of cells monolayer, phagocytose of membrane portions of MDCK^c^ cells, induction of apoptotic bodies and membrane blebbing of HeLa^e^ cells) = evidence of virulence supporting a pathogenic role for *T*. *tenax*. **Limitations**: *in vitro* cellular model not fully reflect clinical reality. Differences between virulence factors of ATCC^a^ and wild clinical strains.
	Alderete *et al*. 1985 [18]	*Adhesion *T*. *tenax* ATCC^a^ (axenic medium.) or *T*. *vaginalis*—Cells monolayer (HEp-2 ^d^, HeLa^e^ and human fibroblast cell lines). * **Interaction**s (MOI^h^ 5:1) *T*. *tenax*- cells, during 30 minutes.	* *T*. *tenax*: no adhesion, no damage to HeLa * *T*. *vaginalis*: adhesion to cell monolayers, cytotoxicity on HeLa^e^	**Outcomes**: no adhesion and toxicity capacity of *T*. *tenax* ATCC (lack of cell adhesion and HeLa^e^ cytotoxicity capacities). **Limitations**: time of contact for test adherence too fast. Differences between virulence factors of ATCC^a^ and wild clinical strains.
	Alderete *et al*. 1984 [19]	*Cytotoxicity: *T*. *tenax* ATCC^a^ (axenic medium) or *T*. *vaginalis* NHY 286 -epithelial cells (HEp-2 ^d^ and HeLa^e^) * **Interaction**s (MOI^h^ 1:1, 5:1), *T*. *tenax*-cells, during 20h. *Evaluation of viability of host cells (trypan blue uptake and release of ^3^H-thymidine of labelled cells)	* No or little cytotoxicity of HeLa^e^ cell monolayer due to *T*. *tenax*. * Extensive disruption of monolayers and irreversible cellular damage due to alive *T*. *vaginalis*.	**Outcomes**: no measurable cytotoxicity on HeLa^e^ cells by *T*. *tenax* = commensal protozoan of normal flora of oral cavity. **Limitations**: *in vitro* study not always reflect clinical reality, cytotoxicity from only one strain of *T*. *tenax*, cell lines no representative of cytotoxicity on primary cell line.
	Ribaux *et al*. 1983 [45]	*Two strains of *T*. *tenax* (one from patient ulcerative gingivitis, other from laboratory, xenic medium) *Research of fibronectin (immunofluorescence technique)	*Fibronectin like protein: localisation on plasma membrane and axostyle. *Areas of intense labelling at contact zones between protozoa and bacteria.	**Outcomes**: capacity to produce fibronectin like protein with potential role in adhesion of *T*. *tenax* to gingival cells, connective tissues and in phagocytosis of bacteria = potential evidence of involvement in microbiota dysbiosis of periodontal diseases. **Limitations**: *in vitro* study of protozoan interactions not always reflect of clinical reality.
**Virulence factors** Enzymes	El Sibaei et al. 2012 [20]	* *T*. *tenax* from patients with periodontitis, gingivitis. *Analysis of protein profile of lysates by SDS-PAGE^i^ and proteinases by non-denaturing gelatin-SDS-PAGE.	*Identification of 19 proteinases bands, among them detection of cysteine-proteinase.	**Outcomes**: capacity of clinical isolate of *T*. *tenax* to have proteolytic activity, putative virulence factor involving in degradation of periodontal tissues. **Limitations**: *in vitro* study not always reflect of clinical reality, microscopic identification of trichomonads without control by PCR^j^ of species.
	Yamamoto *et al*. 2000 [21]	*T. *tenax* ATCC^a^ 30207 (axenic medium). *Identification of enzyme by electrophoresis and effect of enzyme inhibitors	* Identification of cathepsin B-like protein	**Outcomes**: *T*. *tenax* capacity to produce proteinase potentially hydrolysing acid soluble type I collagen, gelatin and participating to degradation of periodontal tissues. **Limitations**: *T*. *tenax* ATCC strain no representative of all virulence factors of clinical wild strains.
	Nagao *et al*. 2000 [22]	*Test of hemolytic activities of *T*. *tenax* ATCC^a^ 30207 (axenic medium) (intact cell, culture supernatant, culture filtrate, cells debris and lipid enriched fractions) on animal and human erythrocytes. * Identification of enzyme with chemical and proteinase inhibitors.	*One hemolysin protein-like (supernatant, filtrate of *T*. *tenax*), inhibited by various cysteine-proteinase inhibitors *Other hemolysin lipid-like (in intact cells, cell-debris, lipid enriched fractions)	**Outcomes**: *T*. *tenax* ATCC capacity to produce hemolysins: potential virulence factor contributing to the periodontal disease process. **Limitations**: *in vitro* study and ATCC strain: no representative of all virulence factors of clinical wild strains.
	Segovic *et al*. 1998 [23]	*T. *tenax* (dental plaque, axenic broth medium.) *Analyse of protozoan lysates	* Major proteolytic activity at acid pH and weak one at basic pH *Identification of different endopeptidases in cell lysates	**Outcomes**: capacity of clinical isolates to synthesize endopeptidases: potential virulence factor in the periodontal disease process **Limitations**: *in vitro* study = not fully representative of mechanisms of patient disease.
	Bozner & Demes 1991 [24]	*Culture filtrate of *T*. *tenax* (private strain, axenic medium). *Analysis of collagen degradation with different inhibitors of *T*. *tenax* proteolysis activity (Electrophoresis)	* Temperature dependent collagenolytic activities on I, III, IV and V collagens. *Involvement of cysteine proteinase in cleavage of collagen.	**Outcomes: c**ollagenolytic activity of *T*. *tenax* = potential virulence factor contributing to degradation of periodontal tissues. **Limitations**: *in vitro* study, and evaluation of enzyme from only one strain: no representative of all virulence factors of clinical wild strains and pathogenic mecanisms in patient.
	Bozner & Demes 1991 [25]	*Lysates and culture filtrate of *T*. *tenax* (private strain, axenic medium). *Test of specific inhibitors on *T*. *tenax* proteolysis activity. *Electrophoretic analysis of *T*. *tenax* proteinases.	*Proteolytic activity of *T*. *tenax*: - cysteine proteinase SH-dependent, pH optimum of 4–7 (from lysate *T*. *tenax* and culture filtrate). - metalloproteinase SH-independent, pH optimum of 8–9 (from lysate *T*. *tenax*).	**Outcomes**: *T*. *tenax* capacity to produce proteinases = potential participation to the periodontal disease process. **Limitations**: *in vitro* study, and evaluation of enzymatic activities from only one strain: no representative of all virulence factors of clinical wild strains and pathogenic mecanisms in patient.
	Ribaux 1979 [55]	*Isolation of *T*. *tenax* from periodontal patients. *Trichomonads grown in xenic medium	*TEM^g^ observation: numerous phagocyted bacteria inside trichomonads cytoplasm. Lysed bacteria in some phagosomes, bacterial division in other.	**Outcomes**: phagocyted bacteria inside cytoplasm trichomonads, some of them stay alive, other are killed. Bacterial degradation by flagellates. Potential synergy between flagellate and host immune cells in fight against bacterial colonization. **Limitations**: no identification of phagocytised bacteria (pathogens or commensal?). No identification by PCR^j^ of trichomonads isolate.
**« Host response »** Innate response	Govro et al. 2016 [26]	*Macrophage (THP-1 cells stimulated) *Hs-4 strain *T*. *tenax* (axenic medium) *MOI^h^ *T*. *tenax*: THP (1:5, 1:10, 1:20 with alive and lysed *T*. *tenax*) during 4,8 & 16h. *Analysis of cytokines (TNFα, IL-1β, IL-8 & IL-10)	* No cytokine production with alive *T*.tenax * Higher IL-8 after 16h with dead *T*. *tenax* (higher MOI^h^, 1:5)	**Outcomes**: no production of cytokines such as TNFα, IL-1β & IL-10 after interactions of macrophage with live or lysed *T*. *tenax*. Synthesis of IL-8 after interaction of macrophage with dead flagellates (MOI^j^, 1:5): evidence of non-pathogenic role of the trichomonads in the etiopathogenesis of periodontitis. **Limitations**: THP-1 cell line not fully representative of reactions of primary culture cells. Need to test other trichomonads as clinical wild strains with different MOI^j^.
**« Host response »** Adaptative host responses	Ioli *et al*. 1987 [27]	* Antigen (centrifugation of cyclic cryolysis laboratory strain of *T*. *tenax*). *Antibody production (administration of *T*. *tenax* antigen in rabbit) *Analysis of *T*.*tenax* antibody in haemodialyzed blood of patients.	* Detection of antibodies against *T*. *tenax* in blood of patients’ haemodialysis	**Outcomes**: identification of *T*. *tenax* antibodies, potential evidence of adaptative host response to trichomonads. **Limitations**: no data regarding the medium (xenic or axenic). No indication of origin of flagellate identified in the blood of patients’ haemodialysis.
	Kott and Adler. 1961 [28]	*T. *vaginalis* and *T*. *hominis* (patient strains, axenic medium). *T. *tenax* (xenic medium). * Intravenous injections of alive flagellate suspension in rabbits. *Agglutination tests	* Detection of 2 types of *T*. *tenax* antibody: -one agglutinin -other which paralysed flagella * No agglutination of *T*. *tenax* antibodies with *T*. *vaginalis* and *T*. *hominis*.	**Outcomes**: identification of *T*. *tenax* antibodies which are not evidence of its pathogenicity because intravenous injection of the flagellate is not an evidence of host response to trichomonads. **Limitations**: No confirmation of *T*. *tenax* capacity to induce antibody production.

ATCC^a^: American Type Cell Collection,

MTT^b^: 3(4-5-dimethylthiazol-2-yl)-2,5-diphenyltetrazolium bromide),

MDCK^c^: Madin-Darby canine kidney =,

HEp-2^d^ (Human epithelial cell),

HeLa^e^: human urogenital and vaginal cells),

SEM^f^ and TEM^g^: Scanning Electronic Microscope and Transmission Electronic Microscope,

MOI^h^: Multiplicity Of Infection (trichomonads: Epithelial cells),

SDS-PAGE^i^: Sodium Dodecyl Sulfate PolyAcrylamide Gel Electrophoresis,

PCR^j^: Polymerase Chain Reaction.

**Table 3:** Extra-oral location of *T*. *tenax*: Research of invasion, colonization and pathogenicity capacity.

Authors	Population- Case Report Medical history	Sample Protozoan identification	Results	Outcomes–Limitations
Brosh-Nisimov *et al*. 2018 [29] Case report series	22 soldiers *T*. *vaginalis* + (8 pharyngeal samples, 14 urines) using Multiplex PCR^a^ tests	*Pharyngeal and urine samples * Anyplex^™^ kit and Xpert-TV^™^ tests To detect false sample *T*. *vaginalis* +	*3/14 (21%) urine samples *T*.*tenax* + with 2 patients with symptoms * 100% pharyngeal samples *T*. *tenax* + Only 1 patient with symptoms	**Outcomes**: presence of *T*. *tenax* in urine = possible role in urogenital infection (patients suffering from dysuria, hematuria, urolithiasis), and presence in pharyngeal sample with only one patient suffering from tonsillitis. Hypothesis of urine contamination from saliva. **Limitations**: no *T*. *tenax* research in saliva to confirm contamination.
Dimasuay and Rivera 2014 [30] Philippines Cohort study	* 44 patients suffering from respiratory ailment	*Sputum *PCR^a^ (amplification of 18S rRNA^b^)	*14 sputums *T*. *tenax* + *1 sputums *T*. *gallinae* +	**Outcomes**: detection of *T*. *vaginalis* in vaginal and *T*. *tenax* in sputum samples. Assumption authors: migration of *T*. *tenax* from oral cavity to the respiratory tract, involvement in aggravation of respiratory infection, no capacity to cause infection. **Limitations**: no research of *T*. *tenax* in oral cavity.
	*186 sex workers	*2 vaginal swab samples /patient *Microscope observation *PCR (amplification of 18S rRNA^b^)	* 19 vaginal swabs *T*. *vaginalis* +	
Leterrier *et al*. 2012 [31] France Case report	**Case report**: woman (67years) * medical history: glioblastoma *After excision of tumoural cyst and corticotherapy, detection of pleural effusion	*Pleural fluid sample *Microscope observation *Culture *PCR^a^	*Identification of *T*. *tenax* and aero-anaerobic flora in sample *Improvement with amoxicillin after metronidazole treatment *Finally, patient died	**Outcomes**: *T*. *tenax* identification in immunocompromised patient (high corticotherapy dose). Assumption authors: possible migration of flagellates to the respiratory tract by aspiration of oropharyngeal secretions. Possible role of co-infecting agent of *T*. *tenax*. **Limitations**: no identification of pathogen bacterial flora in sample.
Crucitti *et al*. 2010 [32] Zambia Cohort study	*460 adolescent girls *307 pregnant women *197 sex workers (= sex)	*Self-collection from vagina, rectum and oral cavity *PCR^a^	*Identification in vaginal infection of: -*T*. *vaginalis*: 24,6% ado, 32,2% pregnant, 33,2 sex -*T*. *tenax*: 0.7% ado, 0.3% pregnant *Identification in oral cavity of: -*T*. *vaginalis*: 0.7% pregnant, 1.1% sex -*T*. *tenax*: 5.3% pregnant, 4.3% sex	**Outcomes**: colonization of pregnant woman and sex workers by *T*. *tenax* and *T*. *vaginalis* both in vaginal and oral samples. Assumption authors: identification of *T*. *tenax* in vaginal swab after autoinoculation via hands or through oral sex. **Limitations**: no report of data about periodontal status of patients and about simultaneous presence of *T*. *tenax* in mouth and vagina.
Chiche *et al*. 2005 [33] France Case report	**Case report**: men (84 years) *medical history: chronic lymphoid leukemia with respiratory and fever * ventilator dependent	*Bronchoalveolar lavage *Microscope observation	* Direct examination of bronchoalveolar lavage was *T*. *tenax* + *Decrease of fever with metronidazole treatment *Finally, patient died	**Outcomes**: putative pathogenicity of trichomonads in older immunocompromised patient. Assumption authors: flagellates colonization of tracheo-bronchial tree after inhalation from oral cavity. **Limitations**: no control of trichomonads’ species by PCR^a^, no research of its presence in oral cavity.
Duboucher *et al*. 2000 [34] France Case report	**Case report**: woman (82 years) *medical history: fever, asthenia and cervical adenopathy *edentate patient without any mucosal wounds nor fistula	*Needle aspiration of cervical lymph node and dental scraping * May-Grünwald-Giemsa staining Microscope observation *Culture of cervical effusion after lymph node excision *Transmission electron microscopy observation.	* Detection of *T*. *tenax* in lymph liquid, cytologic preparation, dental scraping *Identification *Mycobacterium tuberculosis* in culture of excised lymph node. * With metronidazole therapy, healing of cervical lesion, persistence of fever *Finally, patient died	**Outcomes**: detection of *T*. *tenax* in lymph node. Role of *M*. *tuberculosis* (possible role for *T*. *tenax)* in caseous necrosis. Assumption authors: *T*. *tenax* potential invasion of lymphatic vessels after passing through oral mucosa. Possible facilitation of flagellate colonization by tuberculosis. **Limitations**: low-level evidence of invasion and pathogenicity capacity of *T*. *tenax* in immunocompromised patient (case-report). No explanation about origin of *T*. *tenax*. No control of trichomonads’ species by PCR^a^.
Jakobsen *et al*. 1987 [35] Case report	**Case report**: man (52year-old) *medical history: abdominal pain, alcoholic, *subhepatic abscess *partially edentulous	* Aspirated pus from subhepatic abscess. * Microscope observation	*Identification of numerous bacteria and flagellates (*T*. *tenax* or *T*. *hominis)*. *No improvement with intravenous antibiotic (metronidazole + penicillin). *Finally, patient died	**Outcomes**: detection of flagellate microorganism morphological consistent with *T*. *tenax* or *T*. *hominis* in pus and numerous bacteria (*Streptococcus salivarius*, *S*. *milleri*, *Prevotella melanogenica (formerly Bacteroides melanogenicus)* and *B*. *capillosus*. **Limitations**: no PCR^a^ to control trichomonads’ species. Low-level evidence of migration from oral cavity to liver and of pathogenicity of *Trichomonads* (no search of *T*. *tenax* in dental sample).

PCR^a^: Polymerase Chain reaction;

rRNA^b^: ribosomal ribonucleic acid.

### Risk of bias and quality assessment of selected studies and across studies

- **Regarding the case-control studies** (Table 1). For some studies, the absence of detailed protocol of collection of the microbiota makes it difficult to draw any conclusions on the localization of the periodontal colonisation of this protozoan: in sub-gingival and/or supra-gingival biofilm? Even if no study has selected an appropriate sample size of patients to provide sufficient statistical power to detect the flagellate prevalence in periodontitis, studies showing its presence in periodontal pocket could not be discarded. Two of three case-control studies used the microscope to detect *T*. *tenax* which may be biased because its phenotypic characteristics are quite similar to those of *T*. *vaginalis* and *T*. *hominis*, but these species are very rarely described in the mouth [32]. Quality assessment of clinical studies is detailed in S2 Table.
- **Regarding the randomized controlled trials** (Table 1). No periodontal parameters have been established and measurable before and after SRP, periodontal healing cannot be evaluated according to the protozoan decrease. The authors only specified the degree of periodontal degradation with CAL parameter but not the infected periodontal status of the patients (no PPD data). The researcher did not discuss the credibility of their findings with the healing or not of sites with periodontitis, and only reported differences in the frequency of flagellate detection before and after the treatment in both saliva and biofilm sample. S2 Table shows the details of the quality assessment.
- **As regards the laboratory studies** (Table 2). Numerous publications have explored the potential pathogenicity of *T*. *tenax*. However, the authors generally used an ATCC strain that could not fully reflect the virulence of clinical isolates and used cell lines for cytotoxicity testing and cytokine production. Certainly, cell lines can react differently from primary cell lines that constitute the cellular model with the closest similarity to the patient cells. Moreover, the methodological heterogeneity of protocols, the few *T*. *tenax* strains available and the xenic culture medium used in some studies make it difficult to evaluate results. No animal model of periodontitis has been found with *T*. *tenax*.

Medium used in some studies make it difficult to evaluate results. No animal model of periodontitis has been found with *T*. *tenax*.

- **As regards the extra-oral location of *T*. *tenax* to research the pathogenic potential and invasive power** (Table 3). Some studies identified flagellates under the microscope, which is not the most appropriate technique to confirm the trichomonads species. The authors postulated the inhalation of flagellates from the oral cavity to the respiratory tract without any dental sample analysis [30,31,33]. The majority of publications were case reports or case report series; further investigations are required to conclude of any T. tenax pathogenicity in an extra-oral location.

## Discussion

This systematic review has evaluated the role of *T*. *tenax* in the etiopathogenesis of periodontitis using Koch’s postulates revisited by Socransky as PICO framework. The principle findings were discussed for each postulate and concerned in particular two of them: “Association” and “Virulence factors”.

Among the various postulates and causal models that could provide evidence of pathogenicity for a putative periodontal pathogen, Koch’s postulates revisited by Socransky is the most well-known and used. Unfortunately, the implementation of these postulates is limited to cultivable microbiota and has never be adapted to uncultivable microorganisms identified by molecular biology that could also be suspected of initiating or participating in the pathogenesis of the disease.

These Koch’s postulates must be adapted to new technologies and could be based on the detection of nucleic acid sequence as suggested by Fredricks and Relman [36]. But these last postulates cannot be applied to this literature review, because in most of the clinical studies the authors identified *T*. *tenax* using microscopy.

It is increasingly clear that periodontitis is the result of the synergy of microorganisms embedded in a biofilm [37]. But among all the detected microorganisms the Socransky modified postulates remains a way to discriminate pathogenic bacteria in periodontitis. In this review, these postulates are applied to protozoan to compare their virulence factors to those of well-known periodontopathogens.

### The prevalence of *Trichomonas tenax* in periodontitis

Periodontitis is an inflammatory disease resulting from dysbiotic microbial communities of pathobiont and keystone pathogens, which develop virulence factors inducing periodontal tissue degradation. The dysbiosis of periodontal microbiota results in the breakdown of the bacterial balance in favour of periodontopathogens and to the detriment of commensal bacteria [38]. The pathogens are present in relative low abundance in the healthy sites and their quantity is proportional to the PPD in diseased sites.

**The real prevalence of *T*. *tenax* in periodontitis** could not be established for two reasons: (i) the patients sample size seems to be insufficient to achieve a statistical significance, (ii) the periodontal parameters were not detailed enough. Nevertheless, this protozoan has been found in healthy sites as well as sites with gingivitis and periodontitis, from 7.1 to 28.2%, 0 to 32.3% and 32.3 to 42.5% respectively. No indication on the quantity of flagellates in the various sites is known. The implementation of the dysbiosis concept for *T*. *tenax* cannot be totally excluded as the flagellate is detected in both pathological and healthy sites as indicated for periodontal pathogens [7,8,15,39]. The identification method (direct observation or observation of the culture after the incubation time) and the heterogeneity of the sampling protocol make it difficult to conclude on the specific identification of the protozoan in periodontitis due to the morphological similarities with *T*. *hominis* and *T*. *vaginalis*. Nevertheless, one case-control study used the PCR assay method and confirmed the presence of trichomonad species in samples from both patients with or without periodontitis. Some clinical studies have identified the flagellate with PCR, but they could not be included in this review because authors have selected patients with periodontal diseases as manifestation of systemic disease and/or patients without any description of periodontium [40, 41, 32].

### The postulate “Elimination”

**The postulate “Elimination”** evaluates the impact of non-surgical periodontal therapy on the decrease or elimination of a putative pathogen in a healed site. Only one randomly controlled trial evaluates the impact of scaling and root planing (SRP) on the frequency of protozoan identification in patients with periodontitis (CAL > 5 mm) [16]. Surprisingly, the SRP treatment induced a decrease of *T*. *tenax* in saliva but not in dental plaque. Some authors found a high frequency of the flagellate in patients with poor hygiene [42].

One might expect that improved hygiene and a debridement of the periodontal pocket would reduce the number of flagellates, as demonstrated for bacteria [43]. Only one periodontopathogen was not eliminated after SRP: *Aggregatibacter actinomycetemcomitans* [44]. This pathobiont presents numerous virulence factors and a tissue and cell invasiveness capacity that could explain “its resistance” to non-surgical treatment, but these properties have not been described for flagellates. In the absence of periodontal parameters description before and after treatment and in the absence of dental sample location (subgingival and/or supragingival), it is difficult to draw solid conclusions from this study.

### The postulate “Virulence factors”

Numerous authors have evaluated the factors that may respond to the **postulate “Virulence factors”** in relation to *T*. *tenax*. Cellular adhesion is the first step in tissue colonisation for any microorganism. The adhesion and colonisation capacity has clearly been described for *T*. *vaginalis* but not for *T*. *tenax* [18,19]. In 1983, Ribaux et al. described fibronectin-like protein located on plasma membrane and protozoan axostyle, which could potentially have a role in *T*. *tenax* adhesion to eukaryotic cells and in the phagocytosis of bacteria. These authors demonstrated a particularly intense labelling localised in the contact zone between bacteria and two strains of *T*. *tenax* by indirect immunostaining, one isolated from a patient and the other from a laboratory strain, cultured in xenic medium [45]. This aspect was confirmed by Ribeiro et al. who demonstrated the ability of *T*. *tenax* ATCC 30207 to adhere to both cell lines and primary cells (patient epithelial cells) [17].

The explanation of the inconsistencies between the Alderete and Ribeiro adhesion results could be attributed to the different times of protozoan cell interaction: thirty minutes (no contact between the flagellates and cells) and 2 hours (visible interaction between the flagellates and the epithelial cells) respectively [17– 19].

Moreover, Ribeiro has also shown the ability of *T*. *tenax* to induce cytotoxic effects such as damage and disruption of cell monolayers. The oral protozoan appears to phagocytose membrane portions of MDCK (Mardin-Darby Canine Kidney) epithelial cells and induces membrane blebbing and apoptotic bodies in HeLa cells. The epithelial tissue is the first barrier protecting the periodontium. The potential capacity of *T*. *tenax* to induce damage in cell monolayers could promote and facilitate its invasion into the deepest connective tissue. This hypothesis should be confirmed in animal models and with the analysis of gingival tissue of patients with periodontitis. This cytotoxicity would prove the pathogenic role of *T*. *tenax* and its potential participation in the cell breakdown described in periodontal diseases [46]. Likewise, Alderete observed no measurable cytotoxicity due to the too short contact of *T*. *tenax* with epithelial cell lines [18].

Numerous articles dealt with the capacity of *T*. *tenax* to produce enzymes. The authors have identified some of them and assessed their properties. In 1991, Bózner and Demes, and more recently El Sibaei, identified a temperature-dependant proteinase capable of degrading types I, III, IV and V collagen, that would be cysteine proteinases or metallo-proteinases [20,24,25]. These enzymes are cell-associated and extracellular proteinases released from alive *T*. *tenax* [25]. Moreover, Yamamoto et al. have shown that an ATCC *T*. *tenax* strain can produce a specific enzyme that hydrolyses acid soluble type I collagen as well as gelatin, described as cathepsin B [21]. Numerous publications have described one of the main virulence factors in periodontitis: cysteine proteinases, mainly produced by *P*. *gingivalis* but also by *Tannerella forsythia*, are capable of directly degrading host tissues, activating the host proenzymes or neutralizing the host immune system [47,48]. The collagenolytic activities of *T*. *tenax* proteases could also, as indicated for bacterial cysteine proteases, participate in host matrix protein damage and play a role in the etiopathogenesis of periodontal diseases [49]. But Bózner et al. concluded that it would be of interest to purify *T*. *tenax* enzymes and test their effect on extracellular matrix proteins in the oral cavity [25]. In addition to these enzymes, *T*. *tenax* seems to be able to produce haemolysins, which are also synthesised by many pathobionts such as *P*. *gingivalis*, *Prevotella intermedia*, *A*. *actinomycetemcomitans* and *Treponema denticola* [50–53, 22].

### The postulate “Host responses”

All bacteria regarded as periodontal pathogens can trigger a **"Host response"** to both innate and adaptative immunity. The only study assessing the response of macrophages to contamination with live ATCC *T*. *tenax* strain failed to show pro-inflammatory cytokine production [26]. Nevertheless, interleukine-8 is the only macrophage-produced cytokine after 6 hours of incubation with flagellate lysates. The absence of cytokine synthesis with live flagellate could be the result of the low ratio of trichomonads: macrophage, 1:5. Certainly, Ribeiro’s cytotoxicity assay used an inverted ratio (5:1). *T*. *tenax* seems capable of inducing antibody production as shown in the blood analysis of haemodialysis patients [27]. Moreover, Kott and Adler detected two types of antibodies: one targeting the *T*. *tenax* flagella and another an agglutinin, but this result is not relevant to show the potential pathogenicity of *T*. *tenax* [28]. Indeed, intravenous injection of a commensal bacteria could induce the production of systemic antibodies [54]. Ioli et al. have detected antibodies against *T*. *tenax* in the blood of haemodialysis patients without controlling the presence of bone loss around teeth of these patients. So, the conclusion of the flagellate involvement in periodontitis should be cautiously considered. All of these *in vitro* studies provided some possible responses to the question of the pathogenicity of *T*. *tenax*. But these *in vitro* cellular models generally used cell lines and ATCC *T*. *tenax* strain that cannot fully replicate the *in vivo* interaction between the *T*. *tenax* wild-type strain and primary human gingival cells.

### Potential pathogenicity in extra-oral location of *Trichomonas tenax*

*T*. *tenax* is described in the literature as an inhabitant of the oral cavity, but some studies have shown this flagellate in extra-oral locations such as the vagina, the respiratory system, the liver and the lymph node of an immunocompromised patient with severe infectious disease. Most of these articles are case reports which have the lowest level of evidence of all clinical studies and conclude that *T*. *tenax* has no invasive power (*via* bloodstream) or pathogenicity. The authors hypothesized that the flagellate moved from the oral cavity to the lung or was self-inoculated via the hands or through oral sex to the vagina and was only a co-factor aggravating the underlying infection. Duboucher et al. could not explain the lymphatic colonization of the flagellate, although an invasion via the lymphatic vessels, and postulated a putative role in the cervical adenopathy of the patient as this protozoan was the only microorganism identified in the lymph node [34]. Even if three weeks later a culture of the excised lymph node showed *Mycobacterium tuberculosis*, the authors suggest that the observed caseous necrosis could be attributed not only to *M*. *tuberculosis* but also to *T*. *tenax* [34]. Jakobsen speculated about a possible migration through the blood to explain the presence of *T*. *tenax* in subhepatic abscess [35]. Only Brosh-Nissimov et al. have reported the presence of the flagellate in extra-oral locations in immunocompetent patients with urogenital infection. These authors concluded that *T*. *tenax* could be one of the potential etiologies of this dysuria [29].

### Strengths and limitations

As far as is known, this review is the first one to investigate the role of *T*. *tenax* in periodontitis using PRISMA guidelines and Koch’s postulates revisited by Socransky. These results shown an interesting evaluation of the role of this flagellate in the periodontitis of human adult without any systemic nor genetic disease. Nevertheless, this review presents some limitations: (i) language restriction, (ii) limited number of papers with precise periodontal parameters detecting *T*. *tenax*, (iii) majority of the included articles identified this trichomonads by microscope which is not the most accurate technique, (iv) regarding research articles, authors used either ATCC or patient strain which may not be representative of all wild strains in patients with periodontitis (v) and the use of different protocols (MOI, time of incubation, cell lines) makes comparison difficult and does not allow a firm conclusion.

## Conclusions

Even if *T*. *tenax* appears to be identified in patients with periodontitis, there are still many unanswered questions. The cellular model with flagellate has demonstrated its acknowledged adhesion, cytotoxicity and enzyme production that could contribute to periodontal tissue breakdown brought about by periodontopathogens. Its direct pathogenic role in periodontitis but also in extra-oral infections remains unclear and requires further investigations. Periodontal microbiota dysbiosis is not only an imbalance in the relative abundance of certain microbial species found in diseased sites, but also involves polymicrobial synergy leading to immune dysfunctions that result in alveolar bone and soft tissue destruction. In the absence of direct pathogenicity, *T*. *tenax* could be indirectly involved in the etiopathogenesis of periodontitis. Certainly, the bacteria phagocytized by the flagellate may be either lysed or alive inside its cytoplasm and be protected from immune cells and antibiotics action [55]. In this new context, the roles of individual bacteria and their interaction with host and also with other microorganisms such as protozoan must be evaluated.

## Supporting information

S1 Table PRISMA checklist. (DOC)

S2 Table Reasons for exclusion of publications recorded after removal of duplicates. (XLSX)

S3 Table Quality assessment of clinical studies included (based on Newcastle Ottawa Scale). (XLSX)
